# Identification and validation of stage-specific microRNAs and target genes for prostate cancer: Utilizing bioinformatics tools for diagnostic marker discovery

**DOI:** 10.1371/journal.pone.0315366

**Published:** 2025-11-04

**Authors:** Mahsa Yaghobinejad, Mohammad Naji, Ali Mohammad Alizadeh, Soheib Aryanezhad, Solmaz Khalighfard, Parisa Asadollahi, Nasrin Takzare, Tayebeh Rastegar

**Affiliations:** 1 Department of Anatomy, Tehran University of Medical Sciences, Tehran, Iran; 2 Urology and Nephrology Research Center, Shahid Beheshti University of Medical Sciences, Tehran, Iran; 3 Cancer Research Center, Cancer Institute, Tehran University of Medical Sciences, Tehran, Iran; 4 Uro-oncology Research Center, Tehran University of Medical Sciences, Tehran, Iran; 5 Research Center for Developement of Advanced Technologies, Tehran, Iran; 6 Department of Microbiology, Ilam University of Medical Sciences, Ilam, Iran; BMSCE: BMS College of Engineering, INDIA

## Abstract

Given the urgent need for more specific, sensitive, and non-invasive markers for prostate cancer screening and differential diagnosis, circulating miRNAs have emerged as valuable candidates. Sixty seven prostate cancer subjects in different stages were included in this study. The participants were categorized into groups based on their pathological characteristics as local, biochemical relapse and metastatic. We retrieved eligible datasets from GEO database to identify stage-specific differentially expressed up/down-regulated genes. Cytohubba, built-in application of Cytoscape software, and Reactome pathway database were applied to select hub genes. To select upstream miRNAs, we utilized the MiRWalk and miRNet online tools. To construct the miRNA-mRNA regulatory networks, we employed rna22. Finally, three miRNAs and five target genes were validated in peripheral blood mononuclear cells of PCa patients compared with benign prostate hyperplasia. PSA level was also measured using ELISA. Our findings revealed the potential role of PRC1 and UBA52 to be used as biomarkers for the metastatic stage, RCC1 for both biochemical relapse, and metastatic subjects. Furthermore, elevated levels of miR-124-3p and downregulation of miR-133a-3p can be introduced as biochemical relapse stage identifier. We also identified the tumor suppressor role of miR-17-5p, which was associated with higher Gleason scores. We propose PRC1, UBA52, RCC1, miR-124-3p and miR133a-3p as stage-specific PCa identifiers.

## Introduction

Prostate cancer (PCa) is a heterogeneous disease, and this heterogeneity is observed among different PCa stages (localized vs metastatic) [1]. According to the Global Cancer Statistics (GLOBOCAN 2022), PCa is reported as the second most prevalent cancer among men in over half of the countries worldwide, with an increasing mortality rate in Central and Eastern Europe, Asian, and African countries [2]. Although Asian countries have a low incidence of PCa, the mortality rate is increasing in these countries. Meanwhile, age-standardized incidence rates showed an upward trend among individuals aged 20 to 44 [3]. Among various treatment approaches like prostatectomy, chemotherapy, immunotherapy, and radiation therapy [4], androgen deprivation therapy (ADT) is the most commonly used therapeutic strategy for PCa. While most early-stage PCa patients respond to ADT treatment, disease progression leads to the hormone-refractory stage, ultimately resulting in metastasis as metastatic castration-resistant prostate cancer. Commonly used diagnostic tests for PCa detection include serum prostate-specific antigen (PSA) monitoring, digital rectal examination, histopathological findings, and various imaging techniques. However, serum PSA evaluation, although beneficial in early diagnosis, is insufficient for accurate patient risk stratification and lacks sensitivity in PCa diagnosis. It can also be elevated due to benign prostatic hyperplasia (BPH), inflammation, and infection, leading to over-diagnosis and over-treatment [5,6]. According to the silent nature of PCa, most of the patients diagnosed with PCa may have metastatic sites, especially in bones that become a chief problem [7]. Hence, there is an urgent need for more sensitive and stage-specific biomarkers for PCa diagnosis and progression assessment. In the past decade, miRNAs have garnered attention as novel biomarkers for detecting tumor presence, identifying subtypes, predicting therapeutic response, and assessing patient survival due to their accuracy and less invasive accessibility [8–10].

MiRNAs are small noncoding RNA molecules (~22nt) [5] transcribed from their coding genes by RNA polymerase II, resulting in long primary miRNAs (pri-miRNAs). Following cleavage by the RNA polymerase III enzyme DROSHA, they are exported to the cytoplasm through Exportin-5. In the cytoplasm, they undergo processing by DICER, another RNA polymerase III enzyme, to generate mature miRNAs. These mature miRNAs can then interact with their target mRNAs in the cytoplasm or bind to Argonaute 2 protein, a subunit of the RNA-induced silencing complex, where they become single-stranded and active. They can also be encapsulated within microvesicles, exosomes, or bound to apolipoproteins like HDL, and released into the bloodstream [11,12]. Mature single-stranded miRNAs participate in various biological pathways, and their dysregulation is associated with a wide range of diseases, including malignancies [13]. They can be classified as either oncomiRs or tumor-suppressor miRNAs [9], and they exert their effects on target mRNAs by binding to the 3’ untranslated region [14], leading to translation repression and mRNA destruction. Due to their “loose specificity binding” with their targets, miRNAs can regulate the expression of multiple oncogenes and tumor-suppressor genes [12,15]. However, bioinformatics, which integrates mathematics, statistics, and biological findings, facilitates the prediction of potential miRNA-mRNA interactions and their corresponding pathways. Subsequently, experimental techniques can be employed by scientists to evaluate these interactions [13,16]. In our study, we aimed to investigate the expression levels of miR-133a-3p, miR-124-3p, and miR-17-5p, as well as their downstream targets, including heterogeneous nuclear ribonucleoprotein C(HNRNPC), ubiquitin A-52 residue ribosomal protein fusion product 1(UBA52), protein regulator of cytokinesis 1(PRC1), polo-like kinase 1(PLK1), and regulator of chromosome condensation 1(RCC1), which were predicted by our bioinformatics analysis. We conducted qRT-PCR analysis using peripheral blood mononuclear cells (PBMC) obtained from PCa patients at different stages of the malignancy. Our goal was to explore the possibility of utilizing these miRNAs and their downstream targets as stage-specific biomarkers to distinguish between different levels of PCa malignancy.

## Materials and methods

### Meta-analysis based on the GEO database and bioinformatics study overview

A total of 21 datasets (Table 1) were retrieved from the gene expression omnibus (GEO, https://www.ncbi.nlm.nih.gov/geo). Out of these datasets, 15 were specific to the local group, 2 were related to biochemical relapse (BR), 7 were associated with the metastatic group, and 5 were focused on BPH. There were some datasets in common between some groups. Datasets based on PCa cell lines or animal models were excluded from the analysis.

**Table 1:** Details of GEO datasets included in bioinformatics study.

Datasets	Platform	PMID	Groups	Country	Manufacturer
GSE69223	–	26623558	L^a^	Germany	Affymetrix
GSE3325	–	16286247	L, Met^b^	USA	Affymetrix
GSE16120	–	21266046	L	Italy	Agilent Technologies
GSE35988	–	22722839	L	USA	Agilent Technologies
GSE32269	–	23426182	L	USA	Affymetrix
GSE8511	–	–	L, Met	USA	Agilent Technologies
GSE16560	–	20233430	L	USA	Weill Cornell Medical College
GSE6956	–	18245496	L	USA	Affymetrix
GSE68555	GPL92	15254046	L	USA	Affymetrix
GSE119195	–	32392178	BPH^c^	China	Affymetrix
GSE18549	–	–	Met	USA	Affymetrix
GSE5377	–	17646850	L, BPH	Germany	Affymetrix
GSE89243	–	28398479	BR^d^	Lithuania	Agilent Technologies
GSE34933	GPL6480	22703285	BPH	China	Agilent Technologies
GSE89194	GPL22571	28027300	L	USA	Illumina Inc
GSE68882	–	12154061	Met	USA	Affymetrix
GSE33557	–	22915211	BPH	Ireland	Trinity College Dublin
GSE6099	–	17173048	BPH	USA	University of Michigan
GSE6919	GPL92	17430594	L, Met	USA	Affymetrix
	GPL93		L, Met		
	GPL8300		L, Met		
GSE26242	–	–	BR	USA	Illumina Inc
GSE14206	–	22733135	L	Italy	Agilent Technologies

^a^Local.

^b^Metastatic.

^c^Benign prostatic hyperplasia.

^d^Biochemical relapse.

To identify the differentially expressed genes (DEGs) for each stage of PCa progression, we performed a comparison between PCa and noncancerous samples using the GEO2R tool (http://www.ncbi.nlm.nih.gov/geo/geo2r/), which is an online program based on the limma R package. We applied a threshold of P < 0.05 and | log2FC|>1 to determine statistically significant DEGs [17,18]. Prior to conducting the analysis with GEO2R, we conducted grouping based on the histopathological characteristics of the PCa patients, such as clinical stage and Gleason score, as provided in the sample descriptions. Venn diagrams were then generated using FunRich version 3.1.3 to identify the most specific DEGs associated with each stage of PCa progression. Furthermore, we assessed the gene specificity and expression level in PCa using the cancer genome atlas prostate adenocarcinoma (TCGA-PRAD) database available on the University of Alabama at Birmingham cancer data analysis portal (UALCAN) web resource (https://ualcan.path.uab.edu/), which facilitates the analysis of cancer OMICS data.

For the retrieval of interacting genes, we conducted protein-protein interaction analysis and expanded the gene network. Initially, for UP-DEGs, we imported 582 local symbols, 7796 symbols for BPH, 2803 symbols for BR, and 2089 symbols for the metastasis stage. For DOWN-DEGs, we imported 5888 symbols for the local group, 1433 symbols for BPH, 1191 symbols for BR, and 8847 symbols for the metastasis stage. These symbols were separately entered into the Search Tool of the STRING database (https://string-db.org/), and combined scores > 0.4 were considered significant [19]. To identify the top 10 hub genes, we utilized the cytoHubba plug-in in Cytoscape version 3.9.1, with nodes ranked based on their degrees. Additionally, we employed the Reactome pathway database (https://reactome.org/) to uncover significant biological cooperation.

**Fig 1:**
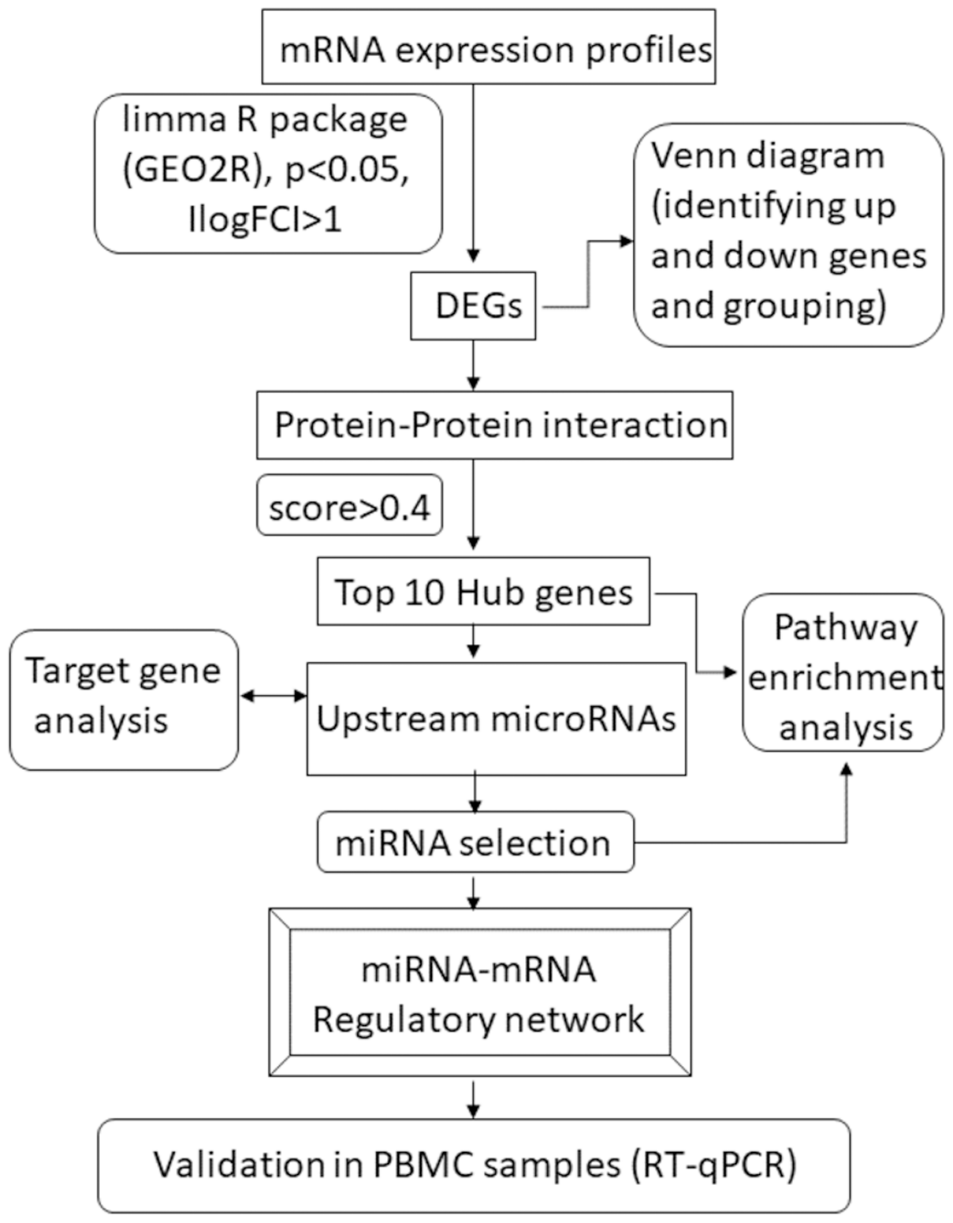
Flowchart of the bioinformatics study. DEG, Differentially Expressed Gene.

To identify the upstream miRNAs, the selected DEGs were analyzed using the miRWalk2.0 database (http://mirwalk.umm.uni-heidelberg.de/). The output from miRWalk was then used to construct miRNA networks by submitting it to the online software miRNet (https://www.mirnet.ca/miRNet/). Additionally, for further investigations regarding miRNA pathway enrichment, we utilized mirPathdeg v.3, an online software based on DIANA tools (https://diana.e-ce.uth.gr/). To extract the exact miRNA-mRNA binding sites, we employed Rna22 (https://cm.jefferson.edu/rna22/) (Fig 1).

### Participants’ enrollment

Blood samples were collected from PCa patients (Table 2) who were receiving care at the Imam Khomeini Cancer Institute and Urology Department in Tehran, Iran. All patients received hormone therapy. However all biochemical relapse subjects got both prostatectomy and hormone therapy. The age range of the patients was between 50 and 88 years, and their Karnofsky Performance Status was equal to or greater than 70. Patients with hypertension, acute infection, tuberculosis, diabetes mellitus, coagulation problems, hepatitis, and autoimmune diseases were excluded from the study. The patients were grouped based on the clinical trials classification for PCa recommended by Scher et al. [20]. Patients with tumors limited to the prostate capsule and identified with T1 to T2 clinical stage were categorized as the local group, patients with tumor growth beyond the prostate capsule identified with T4 clinical stage were categorized as the metastatic group, and patients who experienced PSA elevation to 0.2 ng/ml or higher after radical prostatectomy were categorized as the BR group. A total of sixteen patients diagnosed with BPH through core needle biopsy were included as controls. The clinical data of all patients were obtained from medical reports and informed written consent was obtained from each enrolled patient. This study was approved by the Tehran University of Medical Sciences Research Ethic Committee (IR.TUMS.MEDICINE.REC.1398.456). This study was conducted from 2020/1/21 to 2022/9/1.

**Table 2:** Summarized PCa patient’s characterization.

Characteristic	BPH^a^ (N = 16)	Local (N = 21)	Metastatic (N = 19)	Biochemical relapse (N = 11)
Age- year	62	70.76	69.05	65
BMI, kg/m^2^ ^b^	26.52	26.17	26.27	28.73
Smoking habit				
Yes	2	7	4	2
No	10	8	11	7
Family history				
Yes	19%	15%	32%	28%
Yes, prostate	0%	23%	15%	9%
No	56%	33%	43%	45%
Metastasis (%)				
Soft tissue	–	–	37%	–
Bone only	–	–	87%	–
Both	–	–	25%	–
Tumor stage- no (%) ^c^				
T1	–	0%	–	–
T2	–	66%	–	–
T4	–	–	73%	–
creatinine	0.94	1.28	1.27	1.25
Urea	34	15.5	35.5	32.25
Lobe involvement				
One lobe	–	28%	5%	–
Both lobes	–	28%	73%	–
Treatments				
ADT ^d^	100%	100%	100%	–
ADT + ECTOMY		–	–	100%

^a^Benign prostatic hyperplasia.

^b^Body mass index.

^c^Number.

^a^Androgen deprivation therapy.

### Sample collection, processing, and PBMC extraction

Peripheral blood samples of approximately 10 ml were obtained. Subsequently, 5 ml of the blood sample was transferred into a disposable Golden Vac® EDTA.K2 vacuum blood collection tube for the extraction of PBMCs, while an equal volume was transferred into a Golden Vac® Clot vacuum blood collection tube for serum extraction. The blood samples were kept at a temperature of 4°C until the extraction process, which was performed within a maximum of 2 hours after collection. To isolate the serum, the whole blood was centrifuged at 869 ×  g for 6 minutes. For PBMC extraction approximately 5 ml of the collected blood was mixed by gently inverting the blood collection tube with an equal volume of PBS (1:1). About 4 ml of Ficoll density gradient medium was added to a 15 ml centrifuge tube, followed by gently layering the blood-PBS mixture (1:2) over the Ficoll without disturbing the Ficoll surface by holding the pipette tip alongside the tube wall. Subsequently, the tube was centrifuged at 514 ×  g for 20 minutes at a temperature of 4°C.The PBMC layer at the plasma/Ficoll interface, was carefully collected and transferred into a new centrifuge tube. The collected PBMCs were washed twice with a 1:5 PBS solution and centrifuged at 869 ×  g for 6 minutes at 4°C. After removing the supernatant, the pellet of PBMCs was flash-frozen and stored at -80°C. Throughout the study, all equipment used was RNase-free.

### Total RNA isolation and complementary DNA synthesis

Total RNA was extracted from PBMC samples in each group using RNX-Plus reagent (Sinaclon, Iran) following the instructions provided by the manufacturer. The purification and quantification of the extracted RNA were assessed using a UV spectrophotometer (BIOWAVEΙΙ, England). Finally, the RNA samples were stored at -80ºC until further use. The total extracted RNA was subjected to reverse transcription to synthesize cDNA using the instructions provided by the company (Sinaclon, Iran); random hexamer and stem-loop RT primer were used for cDNA synthesis of mRNA and microRNAs respectively. For miRNA cDNA synthesis, reverse transcription (RT) reactions were performed in a volume of 10 µl, with each reaction containing 50 ng of total RNA. Diethyl pyrocarbonate (DEPC) water was mixed with 1.5 µl of stem-loop RT primer to obtain a total volume of 10 µl. Stem-loop RT primer mixture was heated at 95ºC for 5 minutes for heat denaturation, followed by cooling on ice. Subsequently, 2.5 µl of stem-loop RT primer, 0.5 µl of 10 mM dNTP, 2 µl of 5X reaction buffer, 0.5 µl of Reverse Transcriptase, and 0.5 µl of RNase inhibitor were added to the reaction mixture.

For cDNA synthesis, 500 ng of total RNA was converted into cDNA in a total volume of 20 µl reaction mixture. The reaction mixture included 1 µl of dNTP mix, 4 µl of 5X reaction buffer, 1 µl of Reverse Transcriptase, 1 µl of RNase inhibitor, 0.5 µl of oligo (dT) primer, 0.5 µl of random hexamer, and sufficient DEPC water to obtain a final volume of 20 µl. The reverse transcription process was carried out with incubations at 25ºC, 42ºC, and 85ºC, followed by a final hold at 4ºC.The resulting cDNA samples were stored at -20ºC until further use. Primers and probes were designed using AllelID 6 software (Table 3).

**Table 3:** MiRNA primers and probes for cDNA synthesis and qRT-PCR.

miRNAs	Primer	Sequence (5´-3´)
hsa-miR-17-5p	RT^a^	GGTCGTATGCAAAGCAGGGTCCGAGGTATCCATCGCACGCATC
		GCACTGCATACGACCCTACC
	Forward	TCTCAAAGTGCTTACAGTGC
hsa-miR-124-3p	RT	GGTCGTATGCAAAGCAGGGTCCGAGGTATCCATCGCACGCATCG
		CACTGCATACGACCTTGGCA
	Forward	GTAAGGCACGCGGTGA
hsa-miR-133a-3p	RT	GGTCGTATGCAAAGCAGGGTCCGAGGTATCCATCGCACGCATCG
		CACTGCATACGACCCAGCTG
	Forward	GCTTTGGTCCCCTTCAAC
U6	Forward	GCTTCGGCAGCACATATAC
	Reverse	ATTTGCGTGTCATCCTTGC
	Probe	CAGGGGCCATGCTAATCTTCTCT
Universal TaqMan® probe		TCCATCGCACGCATCGCACT
Universal reverse		AAGCAGGGTCCGAGGT

^a^Reverse Transcriptase.

### Real-time quantitative PCR and miR-qPCR

Toward quantify the mRNA expression, a 20 µl reaction solution was prepared. The reaction mixture contained 0.75 µl of cDNA, 10 µl of 2X RealQ Plus MasterMix Green without ROX (AMPLIQON, Denmark), 0.8 µl of each forward and reverse primer (10 µM) (Table 4), and 7.65 µl of DEPC water.

**Table 4:** Gene primers including their details used for qRT-PCR.

Gene name	Gene Bank Number	Primer sequence(5´-3´)	Product Size	Annealing Temperature
HNRNPC	NM_001077443.2	Fw ^a^: TGACTTTCAACGGGACTATTATG Rev ^b^: TTCCTGATACACGCTGACG	113	53.5
PLK1	NM_005030.6	Fw: TCAACTTCTTCCAGGATCACAC Rev: AGGAGACTCAGGCGGTATG	106	55
PRC1	NM_001267580.2	Fw: TGAGGAGAAGTGAGGTGCTG Rev: CATATTTCCCGAAGGTGATTTAGG	74	51.8
RCC1	NM_001269.6	Fw: TCACACAGCAGCCCTCAC Rev: CTCCAACAGTCCAATCACACC	85	54.8
UBA-52	NM_001321021.1	Fw: TCTGCCGCAAGTGCTATG Rev: AACCACCTTATTTGACCTTCTTC	111	54.3

^a^Forward primer.

^b^Reverse primer.

For miRNA qPCR, a 20 µl reaction solution was prepared as well. The reaction mixture contained 0.75 µl of cDNA, 10 µl of 2X RealQ Plus MasterMix for probe without ROX (AMPLIQON, Denmark), 0.8 µl of forward primer (10 µ M), 0.8 µl of universal reverse primer (10 µ M) (Table 3), 0.5 µl of universal probe (miR-17-5p, miR-124-3p, miR-133a-3p), U6 probe (for U6 internal control), and 7.15 µl of DEPC water. Real-time RT-qPCR was performed using a Rotor-Gene Q instrument (QIAGEN, Germany) with the following optimized cycling program: 15 minutes at 95ºC (hold stage), followed by 40 cycles of 25 seconds at 95ºC and 60 seconds at 60ºC. The gene expression levels were normalized using GAPDH and U6 for mRNA expression levels and microRNAs respectively [21]. The fold changes were calculated using the 2^-ΔCt^ formula. To confirm the specificity of the PCR, the PCR products were analyzed by electrophoresis on 3% agarose gels, and no-template control samples were included. The RT-qPCR assay was performed in triplicates.

### PSA hormonal assay

To isolate the serum, 5 ml of the blood sample was transferred into a Golden Vac® Clot vacuum blood collection tube and centrifuged at 869 ×  g for 6 minutes. The supernatant was collected and stored at -80°C for the PSA ELISA assay. The pre-coated plate with specific antibodies for PSA was provided in the kits (E-EL-H0091, USA). According to the instructions provided in the company datasheet, 100 µl of each standard solution and serum sample was added to the designated wells and incubated for 90 minutes at 37°C. Afterward, 100 µl of biotinylated detection antibody specific for PSA was added to each well. After 60 minutes of incubation, unattached components were aspirated and washed three times. In the next step, Avidin-Horseradish peroxidase conjugate was added and incubated for 30 minutes. Subsequently, 90 µl of substrate reagent was added, causing the wells containing PSA to turn blue. The reaction was terminated with the addition of stop solution, and the optical density was measured at a wavelength of 450 nm. The concentration was then calculated by comparing it to the standard curve.

### Statistical analysis

To assess the data distribution, the Shapiro-Wilk normality test with a significance threshold of P < 0.05 was performed. Mean comparisons between groups with Gaussian distribution were conducted using one-way ANOVA, while the Kruskal-Wallis test was used for groups without normal distribution. Multiple comparison corrections were applied using the two-stage linear step-up procedure of Benjamini, Krieger, and Yekutieli. Receiver Operating Characteristic (ROC) curves were constructed to evaluate the performance of selected genes and miRNAs as stage-specific identifiers, and the area under the curve (AUC) and its 95% confidence interval were calculated. Spearman’s rank analysis was used to examine the correlation between the expression levels of target genes and upstream miRNAs. For two-variable analysis, the Student’s t-test or Mann-Whitney test was utilized. All statistical analyses were performed using GraphPad Prism, version 9.00. The significant differences were presented as: * P < 0.05, **P < 0.01, ***P < 0.001, and ****P < 0.0001.

## Results

### Identification of DEGs for prostate cancer in different stages of PCa malignancy

**Fig 2:**
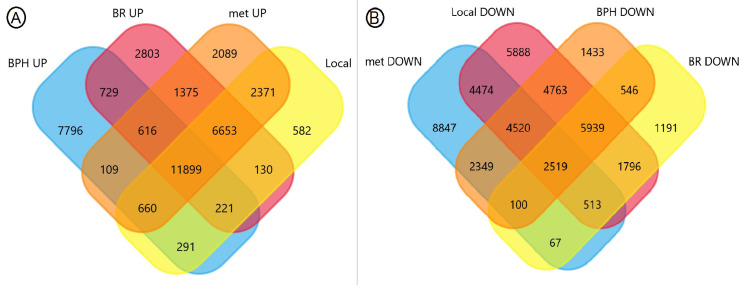
Venn diagrams for UP-DEGs (A) and DOWN-DEGs (B) between different stages of PCa. Using GEO databases, to reach stage-specific target DEGs, genes located in the intersections were omitted. DEG, differentially expressed gene; GEO, Gene expression omnibus; PCa, Prostate cancer; BR, Biochemical relapse; BPH, Benign prostatic hyperplasia; Met, Metastatic.

To obtain DEGs in different stages of PCa, RNA-Seq datasets were extracted from the GEO database. Using the GEO2R analysis tool, comparisons between tumor and non-cancerous samples were performed, resulting in the identification of UP-DEGs and DOWN-DEGs. For the BPH stage, 22 169 DOWN-DEGs and 22 321 UP-DEGs were identified. For the BR stage, 12 671 DOWN-DEGs and 24 426 UP-DEGs were identified. For the local stage, 30 413 DOWN-DEGs and 22 807 UP-DEGs were identified. Lastly, for the metastatic stage, 23 389 DOWN-DEGs and 25 772 UP-DEGs were identified. The significance threshold used was P < 0.05, and the threshold for fold change was | log2FC|>1. Venn diagrams were constructed to identify stage-specific genes and remove redundant ones among them (Fig 2).

### Protein-protein interaction networks

Protein-protein interaction networks were constructed separately for the UP-DEGs and DOWN-DEGs of each group using the STRING database. To identify core protein clusters, the cytoHubba application in Cytoscape software was utilized. The top 10 genes obtained from the analysis were ranked based on their degrees, which represent their connectivity within the network (S1 Table).

Among the UP-DEGs (P < 0.01), three genes were selected: HNRNPC, PLK1, and RCC1. Additionally, two genes were selected from the DOWN-DEGs: PRC1 and UBA52. This selection was finalized according to their high expression level noted in UALCAN web resource and also handling the expenses.

### Pathway enrichment analysis

**Fig 3:**
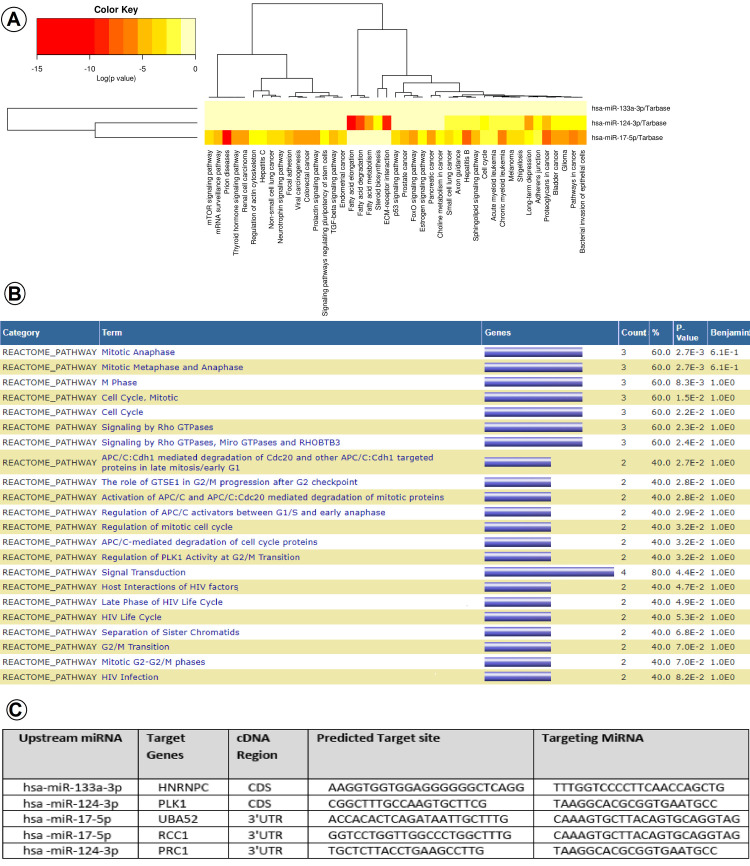
Pathway enrichment analysis and miRNA-mRNA binding sites. (A) MicroRNA pathway heat map constructed with DIANA online tool, mirPath v.3 (P < 0.05). (B) Reactome database functional annotation chart and. (C) miRNA-mRNA interaction sites based on the rna22 web-based tool. CDS, coding sequence; 3’UTR, 3’ untranslated region; HNRNPC, Heterogeneous nuclear ribonucleoprotein C; UBA52, Ubiquitin A-52 residue ribosomal protein fusion product 1; PRC1, Protein regulator of cytokinesis 1; PLK1, Polo-like kinase 1; RCC1, Regulator of chromosome condensation 1.

To identify the biological significance of the selected DEGs, the Reactome pathway database was utilized. The results revealed that the selected DEGs were primarily enriched in various key pathways and biological processes. These included the cell cycle, rRNA major pathway, metabolism of RNA, post-translational protein modification, membrane trafficking, metabolism of lipids and proteins, signaling by hedgehog, mitochondrial protein import, DNA repair, TGF-β receptor signaling cascade, MAPK signaling cascade, and TNF signaling pathway (Fig 3B).

### Identification of upstream miRNAs for prostate cancer based on selected DEGs

To identify upstream miRNAs, the selected DEGs were submitted to the miRWalk web tool, resulting in the retrieval of 6387 miRNAs for HNRNPC, 306 miRNAs for PLK1, 2466 miRNAs for PRC1, 4581 miRNAs for RCC1, and 4857 miRNAs for UBA52. Further analysis was conducted using the miRNet web tool to construct interactional networks (S1–S5 Figs). Among the identified miRNAs, miR-17-5p, miR-124-3p, and miR-133-3p were selected as upstream miRNAs that target the selected DEGs, with statistical significance (P < 0.05).

MiRNA pathway enrichment analysis revealed their involvement in key pathways related to cell cycle, PCa, signal transduction, protein ubiquitin-mediated proteolysis, and other significant pathways associated with malignancies (P < 0.05) (Fig 3A). The selected target genes and miRNAs were further investigated in PBMC samples using RT-qPCR. Additionally, to explore the miRNA-mRNA interactions and binding sites, the selected miRNAs were submitted to the rna22 online web tool (https://cm.jefferson.edu/rna22), yielding valuable insights into the interactions (Fig 3C). The results confirmed that miR-133a-3p targets HNRNPC, miR-124-3p targets both PRC1 and PLK1, and miR-17-5p targets RCC1 and UBA52.

### Gene and miRNA expression analysis and their relationship with PCa levels of malignancy

As mentioned previously, we employed bioinformatics tools to identify stage-specific genes and miRNAs, and investigated their expression levels in PBMC samples across different stages of PCa using qRT-PCR.

**Fig 4:**
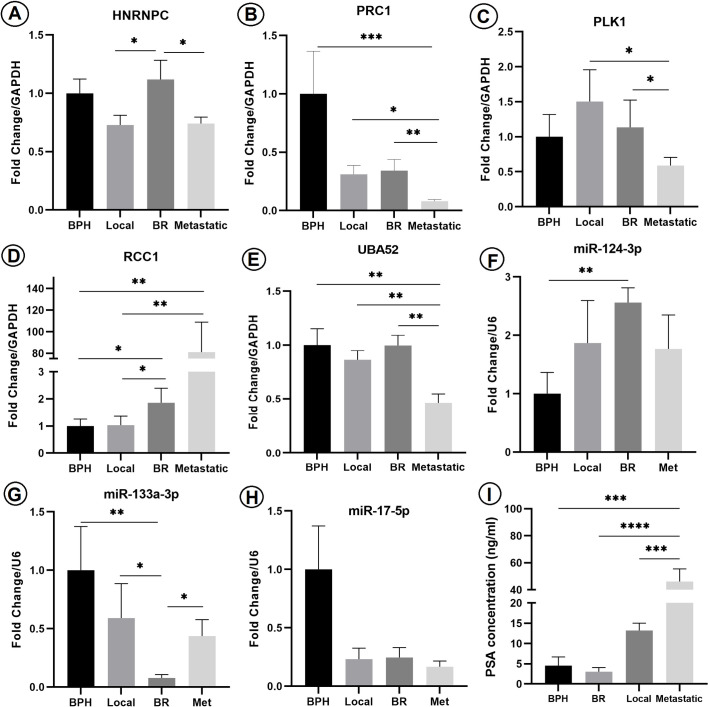
Comparison of the expression level of target genes in PCa PBMCs (A–E). (A) The expression pattern of HNRNPC mRNA. (B) The expression pattern of PRC1. (C) The expression pattern of PLK1. (D) The expression pattern of RCC1. (E) The expression pattern of UBA52. (F) The expression pattern of miR-124-3p. (G) The expression pattern of miR-133a-3p. (H) The expression pattern of miR-17-5p. Kruskal-Wallis test was used for all microRNAs and mRNAs. The concentration level of PSA, one-way ANOVA test (I). * : P < 0.05, **: P < 0.01, ***: P < 0.001 and ****: P < 0.0001. PBMC, Peripheral blood mononuclear cells; PSA, Prostate-specific antigen; BR, Biochemical relapse; BPH, Benign prostatic hyperplasia; Met, Metastatic; HNRNPC, Heterogeneous nuclear ribonucleoprotein C; UBA52, Ubiquitin A-52 residue ribosomal protein fusion product 1; PRC1, Protein regulator of cytokinesis 1; PLK1, Polo-like kinase 1; RCC1, Regulator of chromosome condensation 1.

In the analysis of HNRNPC expression, we observed a significant up-regulation in the BR stage compared to the other two PCa stages (P < 0.05) (Fig 4A). For PRC1, we found lower expression levels in all three PCa stages compared to BPH, with a particularly significant difference between the metastatic and BPH levels (P < 0.001). The metastatic group also exhibited the lowest expression compared to the local (P < 0.05) and BR stages (P < 0.01) (Fig 4B). In the case of PLK1, we observed a significant decreasing pattern across the different PCa stages (P < 0.05). There were significant expression differences between the advanced PCa stages (metastatic and BR) (Fig 4C). RCC1 expression level in metastatic (P < 0.01) and BR stage (P < 0.05) showed a significant increase compared to both BPH and Local stage (Fig 4D).

Regarding UBA52 expression, we found that its expression in the metastatic stage was lower than that in the BPH and the other PCa stages (P < 0.01) (Fig 4E). In terms of miR-133a-3p, we observed the lowest expression level in BR samples compared to other stages (P < 0.05), and it showed a statistical correlation with the BPH group (P < 0.01) (Fig 4G). The expression level of miR-124-3p showed a non-significant upregulation across the different PCa stages, except for a significantly increased expression in the BR patients compared to the BPH (P < 0.01) (Fig 4F). The analysis of miR-17-5p revealed downregulation in the cancer groups compared to the BPH group (P < 0.05), although there was no significant correlation observed between each PCa stage (Fig 4H).

Furthermore, we examined the PSA concentration, which showed a dramatic increase in metastatic samples but did not exhibit any clinico-pathological correlation with the selected target genes and miRNAs (P < 0.05) (Fig 4I).

### Correlation and the predictive value of miRNAs and their downstream target genes for diagnosing PCa

**Fig 5:**
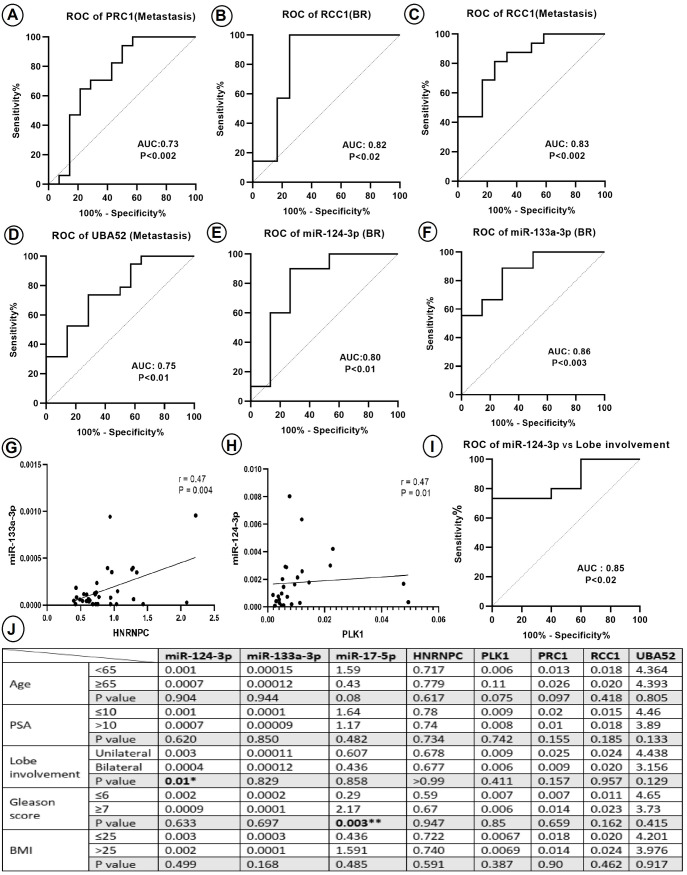
The relationship between gene, microRNAs, and clinico-pathological parameters. According to the ROC curve analysis and calculated AUC the expression level of, (A) PRC1 (CI: 0.54-0.93), (C) RCC1 (CI: 0.69-0.98), and (D) UBA52 (CI: 0.59-0.92) may be identified as biomarkers for metastatic level. (B) The RCC1 (AUC: 0.82, CI: 0.62-1.00), (E) miR-124-3p (CI: 0.62-0.97), and (F) miR-133a-3p (CI: 0.71-1.00) may be identified as biomarkers for BR stage of PCa. Spearman’s correlation analysis showed that (G) HNRNPC expression is correlated with miR-133a-3p expression level (r: 0.47, P = 0.004), (H) PLK1 and miR-124-3p (r: 0.47, P = 0.01) are correlated positively. (I and J) The differential expression of microRNAs and target genes in PBMC samples compared to clinico-pathological features are depicted (Mann Whitney test). * : P < 0.05, **: P < 0.01, ***: P < 0.001 and ****: P < 0.0001. AUC, Area under the curve; ROC, Receiver operating characteristic; AUC, Area under the curve; r, Spearman’s correlation score; CI, Confidence interval; PSA, Prostate-specific antigen; BMI, Body mass index; HNRNPC, Heterogeneous nuclear ribonucleoprotein C; UBA52, Ubiquitin A-52 residue ribosomal protein fusion product 1; PRC1, Protein regulator of cytokinesis 1; PLK1, Polo-like kinase 1; RCC1, Regulator of chromosome condensation 1.

To assess the specificity and sensitivity of the expression levels of the selected genes and miRNAs, we conducted ROC analysis between each PCa stage and its corresponding control group. The calculation of the AUC for the examined genes and miRNAs revealed that PRC1, RCC1, and UBA52 exhibited sensitivity in detecting metastatic PCa, and RCC1 also demonstrated sensitivity in detecting the BR stage. Among the miRNAs, both miR-124-3p and miR-133a-3p showed significant sensitivity as potential markers for the BR stage of PCa (Fig 5A–5F).

To investigate the correlation between the expression levels of miRNAs and their target genes, Spearman’s rank test was conducted. The results indicated a positive relationship between the expression level of HNRNPC and its predicted upstream miR-133a-3p, as well as between PLK1 and its upstream miR-124-3p (Fig 5G–5H).

### Patients’ identifications

There were no significant differences in age, BMI, creatinine, and Urea (obtained from patients’ laboratory reports) between groups. Participants were mainly nonsmokers. According to the patients ‘pathological and bone scan reports, the tumor was mainly invaded to the bones in the metastatic group. Most of the metastatic participants showed both lobe involvement and they had the most positive family history of cancer compared to the other groups. All biochemical relapse participants were received both hormone therapy and prostatectomy and all of the BPH, local and metastatic patients were received just hormone therapy at the time of sampling (Table 2).

### Correlation of PBMC miRNAs and target genes expression level and clinico-pathological parameters

To examine the clinical relevance of the selected miRNAs and their target genes, we investigated the association between their expression levels and the clinico-pathological characteristics of the participants. The analysis revealed a statistically significant relationship between the expression level of miR-124-3p and lobe involvement (P < 0.01). Furthermore, an AUC measurement demonstrated high sensitivity for this finding. Additionally, the expression level of miR-17-5p showed a statistically significant correlation between PBMC samples of PCa patients and the Gleason score (P < 0.003) (Fig 5I and 5J).

## Discussion

We selected and then evaluated three upregulated genes (HNRNPC, PLK1, and RCC1) and two downregulated genes (PRC1, UBA52) that were among the core genes associated with PCa, along with their upstream miRNAs, including miR-124-3p, miR-133a-3p, and miR-17-5p. Consequently, we identified the tumor suppressor role of miR-17-5p, which was related to higher Gleason scores. Pathway enrichment analysis revealed that these five DEGs primarily participated in cell cycle regulation, membrane trafficking, signal transduction, and post-translational protein modification, processes that can undergo alterations during the development of PCa.

The incidence of PCa is steadily rising in Asian countries, with the highest incidence rates observed in countries located in the western region of Asia [22]. The increasing trend among low-incidence countries may be attributed to greater accessibility to diagnostic tests, increased health awareness, socioeconomic status, adoption of a Western-style diet, and genetic predisposition [3]. Integrating computational datasets from GEO and TCGA-PRAD can be valuable in predicting miRNAs and DEGs associated with malignancies. By validating these findings through experimental analysis, they can potentially serve as biomarkers for PCa prediction, classification, and treatment response identification [18]. Moreover, only 2% of human transcriptomes are translated into mRNAs. Non-coding RNAs should be broadly investigated to better understand their role in PCa initiation and progression [23,24]. Through various studies, miR-133a-3p has been recognized as a tumor suppressor in multiple cancers [25–28] and has been proposed as a biomarker for BC diagnosis [29]. Our qRT-PCR results also support this notion by showing a decreased expression level of miR-133a-3p in different PCa stages compared to BPH samples.

Nonetheless, our meta-analysis predicted that miR-133a-3p targets HNRNPC. HNRNPC is a tetramer-shaped protein that belongs to the superfamily of RNA-binding proteins and plays a role in RNA metabolism. Its expression level has frequently been found elevated in various malignancies, including glioblastoma [30], pancreatic cancer [31], PCa [32], non-small cell lung cancer [33], and ovarian cancer [34], indicating its importance in tumorigenesis. However, inconsistent results have been reported for breast cancer [35,36]. Studies investigating exosomes derived from serum samples, using RNA sequencing and qRT-PCR analysis, have shown that HNRNPC is correlated with lymphatic metastasis in PCa [37]. Accumulating evidence suggests that HNRNPC functions as an oncogene, and its elevated expression is associated with tumor growth and metastasis in various cancer types, including PCa [30,32,33,38]. In our bioinformatics analysis, we identified HNRNPC as an upregulated DEG in PCa. However, the qRT-PCR analysis of PBMC samples did not show a statistically significant relationship between HNRNPC expression levels across different PCa stages compared to BPH. This finding is inconsistent with the results reported by Wang et al., which may be attributed to differences in sample types and methodologies applied. Spearman’s rank test confirmed a positive correlation between HNRNPC and miR-133a-3p expression levels. As indicated by ROC curve analysis, the downregulation of miR-133a-3p may contribute to the upregulation of HNRNPC specifically at the BR stage, supporting the potential of miR-133a-3p as a biomarker for the BR stage of PCa.

Polo-like kinase 1 (PLK1) is a serine-threonine kinase that similar to our pathway enrichment analysis, plays a crucial role in cell cycle progression (G2/M transition) and has garnered attention as a potential target for antimitotic drugs [39]. According to Shin et al. the overexpression of PLK1 in PCa is associated with poor survival rates [40].

Based on findings by Yeong et al., using PC3 cell lines, overexpression of PLK1 enhanced resistance and cell survival rates against docetaxel treatment [41]. Although PLK1 expression levels exhibited non-significant upregulation compared to BPH samples, they showed significant downregulation in the metastatic group compared to both the local and BR stages. A study conducted on androgen-insensitive cells (LNCaP-AI) by Deeraksa et al. found that PLK1 was specifically upregulated in LNCaP-AI cells compared to the LNCaP control group [42]. The inconsistency of our results, as explained for PRC1, may be attributed to AR-positive samples or differences in sample types and administration methods.

It is important to note that due to the limited number of samples or variations in sample types, larger-scale experiments will be needed in the future to confirm these findings and provide more conclusive results.

PRC1 is one of the members of the microtubule-associated proteins (MAPs) family that is involved in cytokinesis and its downregulation caused aberrant spindle formation in the central area during the anaphase phase which leads to chromosomal instability and finally tumor evolution [43–45]. A growing body of evidence has shown that PRC1 upregulation is present in different types of malignancies [45]. In M. Shen’s study searching the relationship between PRC1 expression level and PCa metastasis, they found that it would be over-expressed just in double-negative PCa (DNPC) but not in the AR-pathway active (ARPC) and neuroendocrine PCa (NEPC) samples [46]. According to our bioinformatics and mRNA findings we found that PRC1 was downregulated in different PCa levels, especially in Metastasis samples. Moreover, ROC curve findings indicated that it has enough sensitivity to be used as a biomarker for Metastatic group. Based on M. Shen’s results, the inconsistency of our work may be caused by the predominance of ARPC and NEPC samples. According to this study, PRC1 overexpression in response to epigenetic modifications was only detected in DNPC, but not in any other subtypes. According to IHC results presented by Luo et al., the comparison of prostate cancer tissue and normal tissue identified this gene as a biomarker for the recurrence of PCa [47]. Although we identified non-significant upregulation in the BR stage, we observed downregulated expression among cancerous samples. The conflicting findings of this study compared to those of Luo et al. may have arisen from the inclusion of a large number of advanced tissue samples with high pathological stages, as well as a lack of subgrouping of tissue samples. Additionally, the inclusion of a significant number of AR-negative tissue samples in this study may have contributed to these differences.

According to our meta-analysis, PLK1 and PRC1 are predicted to be target genes of miR-124-3p. MiRNA-124-3p participates in different signaling pathways related to tumor cell migration and invasion and its downregulation in PC3 cells was linked to cell proliferation and invasion [48]. MiRNA-124-3p has been recognized as downregulated in various types of cancers, serving as a tumor suppressor molecule, including in PCa [49–52]. However, several studies have pointed to the crucial role of miR-124-3p in maintaining proliferation in PCa [53] and triple-negative breast cancer [54]. By these experiments, our results showed significant upregulation in miR-124-3p expression level in PBMC samples. In line with the other findings, ROC analysis confirmed that the miR-124-3p expression level in the BR stage had the potency to be identified as a biomarker. The clinico-pathological study also revealed a significant relationship between the lobe involvement of PCa samples and miR-124-3p expression level which its expression level can be identified as PCa aggressiveness. Our findings represented the positive correlation between this miRNA expression and PLK1. According to the literature, PRC1 is regulated by PLK1-dependent phosphorylation, and inhibition of PLK1 or direct targeting of PRC1 can lead to its aberrant expression, subsequently causing cytokinesis defects and cancer progression [44]. Based on our bioinformatics study, miR-124-3p targets both PLK1 and PRC1, and its overexpression may result in abnormal expression patterns of these genes. Moreover, these controversial results may be attributed to a positive response to ADT therapy. Large-scale in vivo and in vitro experiments exploring the miR-124-3p/PLK1/PRC1 axis are recommended to clarify the underlying mechanisms. The clinico-pathological study also revealed a significant relationship between lobe involvement in PCa samples and miR-124-3p expression levels, suggesting that its expression level can be identified as an indicator of PCa aggressiveness.

RCC1 is a chromatin-bound guanine-nucleotide releasing factor that functions as a cell cycle regulator and is involved in chromosome condensation during the late S and early M phases of the cell cycle [7,55]. To date, growing evidence has identified RCC1 as a factor in tumorigenesis, particularly in cervical, lung, and breast cancers [56]. Based on the literature it can act as both an oncogene and tumor suppressor especially for gastric cancer [57,58]. According to our bioinformatics study, mRNA results confirmed a significant increase in RCC1 expression across different stages of PCa, particularly at the BR and metastatic levels. Additionally, the ROC analysis indicated that RCC1 may serve as a potential biomarker for the BR and metastatic stages of PCa. Consistent with the earlier studies, our findings also support the concept that RCC1 may act as an oncogene in PCa.

UBA52 is involved in ribosome ubiquitination, and its knockdown can lead to cell cycle arrest. Although there is limited evidence regarding the exact role of UBA52 in PCa, some studies have reported its upregulation in pancreatic cancer and its association with the promotion and progression of multiple myeloma and gastric cancer [16,59,60]. Khan MW et al. proposed UBA52 as an upregulated gene in cancerous cells while constructing PPI networks [61]. Analyzing ChIP-Seq data for PCa published by Zhang et al., in alignment with our bioinformatics results, UBA52 was identified as one of the highly connected genes in PCa [62]. Through our mRNA results, we identified non-significant downregulation of UBA52. Consistent with our bioinformatics findings, UBA52 exhibited significant downregulation specifically in the metastatic stage of PCa. The AUC analysis confirmed that this downregulation in the metastatic stage has the sensitivity to be considered as a potential biomarker. While our bioinformatics findings align with those of Zhang et al., the inconsistency with Mehwish et al. may be attributed to different sample types. Hence, further experiments on PCa subgroups with larger sample sizes are necessary to explore these findings in more detail.

As predicted by our bioinformatics study, miR-17-5p was identified as an upstream regulator of both RCC1 and UBA52 genes. MiR-17-5p has been characterized as both an oncogene and a tumor suppressor by targeting over 20 genes involved in the G1/S-phase transition of the cell cycle, but its role in PCa remains controversial [16]. It has been referred to as an “alarmiR” due to its elevated levels in serum or plasma, which can serve as a general indicator of tumor pathogenesis [17]. Gong et al. explored the miR-17-5p/PCAF relationship in several PCa cell lines and identified an elevated level of PCAF associated with the downregulation of miR-17-5p. As a result of this interaction, AR was activated, leading to PCa cell growth [63]. Thus, Gong et al. highlighted the inhibitory role of miR-17-5p in suppressing PCa growth. Our findings also demonstrated significant downregulation of miR-17-5p in different stages of PCa compared to the BPH level, although this difference did not reach statistical significance. Furthermore, we did not observe a statistical correlation between miR-17-5p and its predicted target genes. However, the clinico-pathological analysis revealed a statistically significant relationship between miR-17-5p expression and the Gleason score.

## Conclusions

In summary, our study identified five key genes (HNRNPC, PLK1, PRC1, RCC1, and UBA52) and their upstream miRNAs (miR-124-3p, miR-133a-3p, and miR-17-5p) through bioinformatics analysis, which play important roles in cell cycle regulation, signal transduction, and PCa identification. Subsequent qRT-PCR experiments on PBMC samples provided further evidence of the potential of PRC1 and UBA52 as biomarkers for the metastatic stage of PCa, RCC1 as a biomarker for both BR and metastatic stages, and indicated the prognostic value of elevated miR-124-3p and downregulated miR-133a-3p in the BR stage. Our study also revealed the tumor suppressor role of miR-17-5p, which showed a statistically significant association with higher Gleason scores. Additionally, miR-124-3p was identified as an oncogene, and its expression was found to be associated with lobe involvement. However, it is important to note that further experiments with larger and more diverse sample sizes are recommended to validate these results and provide more robust conclusions.

## Supporting information

S1 Table Up and Down DEGs output, obtained from bioinformatics analysis. (DOCX)

S1 Fig MiRNet output for HNRNPC, miR-17-5p is identified as hub microRNA for HNRNPC. (TIF)

S2 Fig MiRNet output for PLK1, miR-124-3p is identified as hub microRNA for PLK1. (TIF)

S3 Fig MiRNet output for PRC1, miR-124-3p is identified as hub microRNA for PRC1. (TIF)

S4 Fig MiRNet output for UBA52, miR-124-3p is identified as hub microRNA for UBA52. (TIF)

S5 Fig MiRNet output for RCC1, miR-17-5p is identified as hub microRNA for RCC1. (TIF)

S1 File Mini dataset. (RAR)

## Abbreviations

ADT: Androgen Deprivation Therapy
ARPC: AR-pathway active Prostate Cancer
AUC: Area Under the Curve
BPH: Benign Prostatic Hyperplasia
BR: Biochemical Relapse
DEG: Differentially Expressed Gene
DEPC: Diethyl Pyrocarbonate
DNPC: Double-negative Prostate Cancer
GEO: Gene Expression Omnibus
HNRNPC: Heterogeneous Nuclear Ribonucleoprotein C
NEPC: Neuroendocrine Prostate Cancer
PBMC: Peripheral Blood Mononuclear Cell
PCa: Prostate Cancer
PLK1: Polo like Kinase 1
PRC1: Protein Regulator of Cytokinesis 1
PSA: Prostate Specific Antigen
RCC1: Regulator of Chromosome Condensation 1
ROC: Receiver Operating Characteristic
TCGA-PRAD: The Cancer Genome Atlas Prostate Adenocarcinoma
UALCAN: The University of Alabama at Birmingham cancer data analysis portal
UBA52: Ubiquitin A-52 residue ribosomal protein fusion product 1

## References

[1] Mateo J, Seed G, Bertan C, Rescigno P, Dolling D, Figueiredo I, et al. Genomics of lethal prostate cancer at diagnosis and castration resistance. J Clin Invest. 2020;130(4):1743–51. doi: 10.1172/JCI132031 31874108 PMC7108902

[2] Hashemi M, Taheriazam A, Daneii P, Hassanpour A, Rezaei S, Hejazi E. Targeting PI3K/Akt signaling in prostate cancer therapy. Journal of cell communication and signaling. 2022:1–21. 36367667 10.1007/s12079-022-00702-1 PMC10409967

[3] Jiang J, Chen B, Tang B, Yang J, Zhang T, Li J, et al. Trends of prostate cancer morbidity in low-Incidence countries from 1990-2019. Cancer epidemiol biomarkers prev. 2024;33(2):186–95. doi: 10.1158/1055-9965.EPI-23-1034 38317630 PMC10844848

[4] Verma S, Pandey M, Shukla GC, Singh V, Gupta S. Integrated analysis of miRNA landscape and cellular networking pathways in stage-specific prostate cancer. PLoS One. 2019;14(11):e0224071. doi: 10.1371/journal.pone.0224071 31756185 PMC6874298

[5] He S, Shi J, Mao J, Luo X, Liu W, Liu R, et al. The expression of miR-375 in prostate cancer: A study based on GEO, TCGA data and bioinformatics analysis. Pathol Res Pract. 2019;215(6):152375. doi: 10.1016/j.prp.2019.03.004 30879885

[6] Srivastava A, Goldberger H, Dimtchev A, Ramalinga M, Chijioke J, Marian C, et al. MicroRNA profiling in prostate cancer--the diagnostic potential of urinary miR-205 and miR-214. PLoS One. 2013;8(10):e76994. doi: 10.1371/journal.pone.0076994 24167554 PMC3805541

[7] Aobchey P, Utama K, Niamsup H, Sangthong P. Gene expression analysis of RCC1, VAV2, RPA3, and SRPK1 for human cervical cancer biomarkers. Gene reports. 2022;26:101445. doi: 10.1016/j.genrep.2021.101445

[8] Zhang X, Li Z, Xuan Z, Xu P, Wang W, Chen Z, et al. Novel role of miR-133a-3p in repressing gastric cancer growth and metastasis via blocking autophagy-mediated glutaminolysis. J Exp Clin Cancer Res. 2018;37(1):320. doi: 10.1186/s13046-018-0993-y 30572959 PMC6302516

[9] Zhang L, Chen X, Liu B, Han J. MicroRNA-124-3p directly targets PDCD6 to inhibit metastasis in breast cancer. Oncol Lett. 2018;15(1):984–90. doi: 10.3892/ol.2017.7358 29387242 PMC5769374

[10] Fang Y, Xu C, Fu Y. MicroRNA-17-5p induces drug resistance and invasion of ovarian carcinoma cells by targeting PTEN signaling. J Biol Res (Thessalon). 2015;22(1)1-10. doi: 10.1186/s40709-015-0035-2 26500892 PMC4619013

[11] Zhang W, Zhang G, Zhao R, Gao S. The potential diagnostic accuracy of circulating microRNAs for prostate cancer: A meta-analysis. Actas Urológicas Españolas (English Edition). 2022 10.1016/j.acuroe.2021.05.005 35260368

[12] Bobbili MR, Mader RM, Grillari J, Dellago H. OncomiR-17-5p: alarm signal in cancer?. Oncotarget. 2017;8(41):71206. 29050357 10.18632/oncotarget.19331 PMC5642632

[13] Riffo-Campos ÁL, Riquelme I, Brebi-Mieville P. Tools for sequence-based miRNA target prediction: What to choose?. Int J Mol Sci. 2016;17(12):1987. doi: 10.3390/ijms17121987 27941681 PMC5187787

[14] Xu Y, Zhang L, Xia L, Zhu X. MicroRNA-133a-3p suppresses malignant behavior of non-small cell lung cancer cells by negatively regulating ERBB2. Oncol Lett. 2021;21(6):1-10. doi: 10.3892/ol.2021.12718 33907567 PMC8063298

[15] Cochetti G, de Vermandois J, Maulà V, Giulietti M, Cecati M, Del Zingaro M, . Role of miRNAs in prostate cancer: Do we really know everything?. Urologic oncology: seminars and original investigations. 2020 10.1016/j.urolonc.2020.03.007 32284256

[16] Saadoune C, Nouadi B, Hamdaoui H, Chegdani F, Bennis F. Multiple myeloma: Bioinformatic analysis for identification of key genes and pathways. Bioinform Biol Insights. 2022;16.doi: 10.1177/11779322221115545 35958298 PMC9358573

[17] Li D, Hao X, Song Y. Identification of the key MicroRNAs and the miRNA-mRNA regulatory pathways in prostate cancer by bioinformatics methods. BioMed Research International. 2018. 10.1155/2018/6204128 PMC6031162 30027097

[18] Liu H, Li L, Fan Y, Lu Y, Zhu C, Xia W. Construction of potential gene expression and regulation networks in prostate cancer using bioinformatics tools. Oxid Med Cell Longev. 2021. doi: 10.1155/2021/8846951 34512870 PMC8426106

[19] Sun J, Li S, Wang F, Fan C, Wang J. Identification of key pathways and genes in PTEN mutation prostate cancer by bioinformatics analysis. BMC Med Genet. 2019;20(1):191. doi: 10.1186/s12881-019-0923-7 31791268 PMC6889628

[20] Scher HI, Halabi S, Tannock I, Morris M, Sternberg CN, Carducci MA, et al. Design and end points of clinical trials for patients with progressive prostate cancer and castrate levels of testosterone: recommendations of the prostate cancer clinical trials working group. J Clin Oncol. 2008;26(7):1148–59. doi: 10.1200/JCO.2007.12.4487 18309951 PMC4010133

[21] Naji M, Aleyasin A, Nekoonam S, Arefian E, Mahdian R, Amidi F. Differential expression of miR-93 and miR-21 in granulosa cells and follicular fluid of polycystic ovary syndrome associating with different phenotypes. Sci Rep. 2017;7(1):1-14. doi: 10.1038/s41598-017-13250-1 29116087 PMC5676684

[22] Luo L, Jiang J, Luan H, Zi H, Zhu C, Li B. Spatial and temporal patterns of prostate cancer burden and their association with socio‐demographic index in Asia, 1990–2019. The Prostate. 2022;82(2):193–202. 34662930 10.1002/pros.24258

[23] Vahabzadeh G, Khalighfard S, Alizadeh AM, Yaghobinejad M, Mardani M, Rastegar T, et al. A systematic method introduced a common lncRNA-miRNA-mRNA network in the different stages of prostate cancer. Front Oncol. 2023;13:1142275. 37251950 10.3389/fonc.2023.1142275 PMC10215985

[24] Vahabzadeh G, Khalighfard S, Alizadeh AM, Yaghobinejad M, Mardani M, Rastegar T, et al. A systematic method introduced a common lncRNA-miRNA-mRNA network in the different stages of prostate cancer. Front Oncol. 2023;13:1142275. 37251950 10.3389/fonc.2023.1142275 PMC10215985

[25] Xu Y, Zhang L, Xia L, Zhu X. MicroRNA-133a-3p suppresses malignant behavior of non-small cell lung cancer cells by negatively regulating ERBB2. Oncol Lett. 2021;21(6):457. 33907567 10.3892/ol.2021.12718 PMC8063298

[26] Kong B, Zhao S, Kang X, Wang B. MicroRNA-133a-3p inhibits cell proliferation, migration and invasion in colorectal cancer by targeting AQP1. Oncol Lett. 2021;22(3):649. 34386071 10.3892/ol.2021.12910 PMC8298993

[27] Weber D, Amar L, Gödde D, Prinz C. Extensive screening of microRNA populations identifies hsa-miR-375 and hsa-miR-133a-3p as selective markers for human rectal and colon cancer. Oncotarget. 2018;9(43):27256. 29930763 10.18632/oncotarget.25535 PMC6007480

[28] Tang Y, Pan J, Huang S, Peng X, Zou X, Luo Y, et al. Downregulation of miR-133a-3p promotes prostate cancer bone metastasis via activating PI3K/AKT signaling. J Exp Clin Cancer Res. 2018;37:1–16. 30021600 10.1186/s13046-018-0813-4 PMC6052526

[29] Bitaraf A, Babashah S, Garshasbi M. Aberrant expression of a five‐microRNA signature in breast carcinoma as a promising biomarker for diagnosis. J Clin Lab Anal. 2020;34(2):e23063. 10.1002/jcla.23063 PMC7031575 31595567

[30] Wang L-c, Chen S-H, Shen X-l, Li D-C, Liu H-y, Ji Y-l, et al. M6A RNA methylation regulator HNRNPC contributes to tumorigenesis and predicts prognosis in glioblastoma multiforme. Front Oncol. 2020;10:536875. 33134160 10.3389/fonc.2020.536875 PMC7578363

[31] Yang N, Liu L, Liu X, Chen Y, Lu J, Wang Z. hnRNPC promotes malignancy in pancreatic cancer through stabilization of IQGAP3. Biomed Res Int. 2022;2022(1):6319685. 35355828 10.1155/2022/6319685 PMC8958073

[32] Wang S, Xu G, Chao F, Zhang C, Han D, Chen G. HNRNPC promotes proliferation, metastasis and predicts prognosis in prostate cancer. Cancer Manag Res. 2021:7263–76. 34584453 10.2147/CMAR.S330713 PMC8464311

[33] Yan M, Sun L, Li J, Yu H, Lin H, Yu T, et al. RNA-binding protein KHSRP promotes tumor growth and metastasis in non-small cell lung cancer. J Exp Clin Cancer Res. 2019;38:1–17. 31775888 10.1186/s13046-019-1479-2 PMC6882349

[34] Park YM, Hwang SJ, Masuda K, Choi K-M, Jeong M-R, Nam D-H, et al. Heterogeneous nuclear ribonucleoprotein C1/C2 controls the metastatic potential of glioblastoma by regulating PDCD4. Mol Cell Biol. 2012;32(20):4237–44. 22907752 10.1128/MCB.00443-12 PMC3457347

[35] Fung PA, Labrecque R, Pederson T. RNA-dependent phosphorylation of a nuclear RNA binding protein. Proc Natl Acad Sci. 1997;94(4):1064–8. 9037006 10.1073/pnas.94.4.1064 PMC19744

[36] Sarbanes SL, Le Pen J, Rice CM. Friend and foe, HNRNPC takes on immunostimulatory RNA s in breast cancer cells. EMBO J. 2018;37(23):e100923. 10.15252/embj.2018100923 PMC6276875 30389667

[37] Chen C, Lin T, Huang J. PD59-10 exosomal HNRNPC promotes the lymphatic metastasis of prostate cancer. J Urol. 2020;203:e1208-e9.

[38] Mo L, Meng L, Huang Z, Yi L, Yang N, Li G. An analysis of the role of HnRNP C dysregulation in cancers. Biomark Res. 2022;10(1):19. 35395937 10.1186/s40364-022-00366-4 PMC8994388

[39] Javed A, Özduman G, Altun S, Duran D, Yerli D, Özar T, et al. Mitotic kinase inhibitors as therapeutic interventions for prostate cancer: evidence from In vitro studies. Endocr metab immune disord drug targets. 2023;23(14):1699–712. doi: 10.2174/1871530323666230303092243 36872354

[40] Shin S-B, Woo S-U, Yim H. Cotargeting Plk1 and androgen receptor enhances the therapeutic sensitivity of paclitaxel-resistant prostate cancer. Ther Adv Med Oncol. 2019;11. doi: 10.1177/1758835919846375 31156720 PMC6515847

[41] Pu YS, Huang CY, Wu HL, Wu JH, Su YF, Yu CTR, et al. EGFR‐mediated hyperacetylation of tubulin induced docetaxel resistance by downregulation of HDAC6 and upregulation of MCAK and PLK1 in prostate cancer cells. Kaohsiung J Med Sci. 2024;40(1):23–34. 37916740 10.1002/kjm2.12766 PMC11895646

[42] Deeraksa A, Pan J, Sha Y, Liu X-D, Eissa NT, Lin S-H, et al. Plk1 is upregulated in androgen-insensitive prostate cancer cells and its inhibition leads to necroptosis. Oncogene. 2013;32(24):2973–83. 22890325 10.1038/onc.2012.309 PMC3499666

[43] Bu H, Li Y, Jin C, Yu H, Wang X, Chen J, et al. Overexpression of PRC1 indicates a poor prognosis in ovarian cancer. Int J Oncol. 2020;56(3):685–96. doi: 10.3892/ijo.2020.4959 31922238 PMC7010224

[44] Li J, Dallmayer M, Kirchner T, Musa J, Grünewald TGP. PRC1: Linking cytokinesis, chromosomal instability, and cancer evolution. Trends Cancer. 2018;4(1):59–73. doi: 10.1016/j.trecan.2017.11.002 29413422

[45] Chen X, Hou D, Dong S, Ai X, Qin Z, Li H, et al. A pan-cancer analysis of the oncogenic role of protein regulator of cytokinesis 1 (PRC1) in human tumors. 2022.

[46] Shen MM. A positive step toward understanding double-negative metastatic prostate cancer. Cancer Cell. 2019;36(2):117–9. doi: 10.1016/j.ccell.2019.07.006 31408617

[47] Luo H-W, Chen Q-B, Wan Y-P, Chen G-X, Zhuo Y-J, Cai Z-D, et al. Protein regulator of cytokinesis 1 overexpression predicts biochemical recurrence in men with prostate cancer. Biomed Pharmacother. 2016;78:116–20. doi: 10.1016/j.biopha.2016.01.004 26898432

[48] Fasoulakis Z, Daskalakis G, Diakosavvas M, Papapanagiotou I, Theodora M, Bourazan A, et al. MicroRNAs determining carcinogenesis by regulating oncogenes and tumor suppressor genes during cell cycle. Microrna. 2020;9(2):82–92. doi: 10.2174/2211536608666190919161849 31538910 PMC7366009

[49] Xue M, Li Y, Hu F, Jia Y-J, Zheng Z-J, Wang L, et al. High glucose up-regulates microRNA-34a-5p to aggravate fibrosis by targeting SIRT1 in HK-2 cells. Biochem Biophys Res Commun. 2018;498(1):38–44. 10.1016/j.bbrc.2017.12.048 29371016

[50] Du Y, Wei N, Hong J, Pan W. Long non-coding RNASNHG17 promotes the progression of breast cancer by sponging miR-124-3p. Cancer Cell Int. 2020;20:1–9. 10.1186/s12935-020-1129-y PMC7003346 32042267

[51] Wang J-R, Liu B, Zhou L, Huang Y-X. MicroRNA-124-3p suppresses cell migration and invasion by targeting ITGA3 signaling in bladder cancer. Cancer Biomark. 2019;24(2):159–72. 10.3233/CBM-182000 PMC13082488 30614803

[52] Shi X-B, Xue L, Ma A-H, Tepper CG, Gandour-Edwards R, Kung H-J, et al. Tumor suppressive miR-124 targets androgen receptor and inhibits proliferation of prostate cancer cells. Oncogene. 2013;32(35):4130–8. 10.1038/onc.2012.425 PMC4111479 23069658

[53] Yan K, Hou L, Liu T, Jiao W, Ma Q, Fang Z, et al. lncRNA OGFRP1 functions as a ceRNA to promote the progression of prostate cancer by regulating SARM1 level via miR-124-3p. Aging (Albany NY). 2020;12(10):8880. 10.18632/aging.103007 PMC7288971 32428870

[54] Yang W, Cui G, Ding M, Yang M, Dai D. MicroRNA‐124‐3p. 1 promotes cell proliferation through Axin1‐dependent Wnt signaling pathway and predicts a poor prognosis of triple‐negative breast cancer. J Clin Lab Anal. 2020;34(7):e23266. 10.1002/jcla.23266 PMC7370722 32125723

[55] Riahi A, Radmanesh H, Schürmann P, Bogdanova N, Geffers R, Meddeb R, et al. Exome sequencing and case-control analyses identify RCC1 as a candidate breast cancer susceptibility gene. Int J Cancer. 2018;142(12):2512–7. doi: 10.1002/ijc.31273 29363114

[56] Jing L, Kwok HF. The intricate roles of RCC1 in normal cells and cancer cells. Biochem Soc Trans. 2022;50(1):83–93. doi: 10.1042/BST20210861 35191966

[57] Ren X, Jiang K, Zhang F. The multifaceted roles of RCC1 in tumorigenesis. Front Mol Biosci. 2020;7:225. doi: 10.3389/fmolb.2020.00225 33102517 PMC7522611

[58] Suraweera A, Brown JAL, Lim YC, Lavin MF. Editorial: Cancer Therapeutics: Targeting DNA Repair Pathways. Front Mol Biosci. 2022;9. doi: 10.3389/fmolb.2022.858514 35242815 PMC8886143

[59] Tian X, Ju H, Yang W. An ego network analysis approach identified important biomarkers with an association to progression and metastasis of gastric cancer. J Cell Biochem. 2019;120(9):15963–70. doi: 10.1002/jcb.28873 31081222

[60] Jovic D, Liang X, Zeng H, Lin L, Xu F, Luo Y. Single-cell RNA sequencing technologies and applications: A brief overview. Clin Transl Med. 2022;12(3):e694. doi: 10.1002/ctm2.694 35352511 PMC8964935

[61] Khan MW, Shams Malick RA, Cherifi H. Discovering Disease Genes in PPI Networks: A Bridge from Centrality to Communities. bioRxiv. 2023:2023.09. 08.556873.

[62] Zhang Y, Huang Z, Zhu Z, Liu J, Zheng X, Zhang Y. Network analysis of ChIP-Seq data reveals key genes in prostate cancer. Eur J Med Res. 2014;19(1):47. doi: 10.1186/s40001-014-0047-7 25183411 PMC4171560

