# Supplementary material for: Identification and validation of stage-specific microRNAs and target genes for prostate cancer: Utilizing bioinformatics tools for diagnostic marker discovery
Source: PLoS One. 2025 Nov 4;20(11):e0315366. doi: 10.1371/journal.pone.0315366 (PMC12585097; doi:10.1371/journal.pone.0315366)
Supplement: S1 Table — (DOCX) [file pone.0315366.s001.docx]

**Table S1. Up and Down DEGs output, obtained from bioinformatics analysis.**

| **Groups** | **Up-regulated DEGs** | **Down-regulated DEGs** |
| --- | --- | --- |
| BPH | ANAPC10, ADORA3,BTBD6, ALDH1A1, BAG3, AKR1C3, ARFGAP3, ANXA5, BRF2, ALDH3A2 | PDYN,ADORA3,MAPK3,MYOD1, CDK1, DUX4L8, RTP5, ADR8K1,KRTP10-1, KRTP10-5 |
| Local | SUMO1, HNRNPR, HNRNPL, HNRNPH1, **HNRNPC**, HNRNPU, SRRM1, DDX39A, LUC7L3, ORC1 | RTP5, CDK1, TAS2R50, TAS2R5, S5orf15, KRTAP10-5, MT-ND2, MT-ND3, MT-ND4, MT-ATP6 |
| Biochemical relapse | RPL39L, RPL13,EIF3K,RPSA, RPS17, GNL3, HGH1, MROH1, KIAA1875, ADCY6 | KIAA0020,NHP2L1, SRSF7, SUGP1, HGH1,RTP5, TUBB4B, SLC25A4, SLC25A6, ADRBK1 |
| Metastatic | HNRNPM,HNRNPD,NAA50,NAA16,PSMD1, UBA3, ISG15, RAB11A, **PLK1**, **RCC1** | COPG2, RGPD5, PTMA, CCL4L1, GPR183, ADORA3, TRA2B, **PRC1**, SRSF10, **UBA52** |
