# Supplementary figures and images for: Identification and validation of stage-specific microRNAs and target genes for prostate cancer: Utilizing bioinformatics tools for diagnostic marker discovery
Source: PLoS One. 2025 Nov 4;20(11):e0315366. doi: 10.1371/journal.pone.0315366 (PMC12585097; doi:10.1371/journal.pone.0315366)

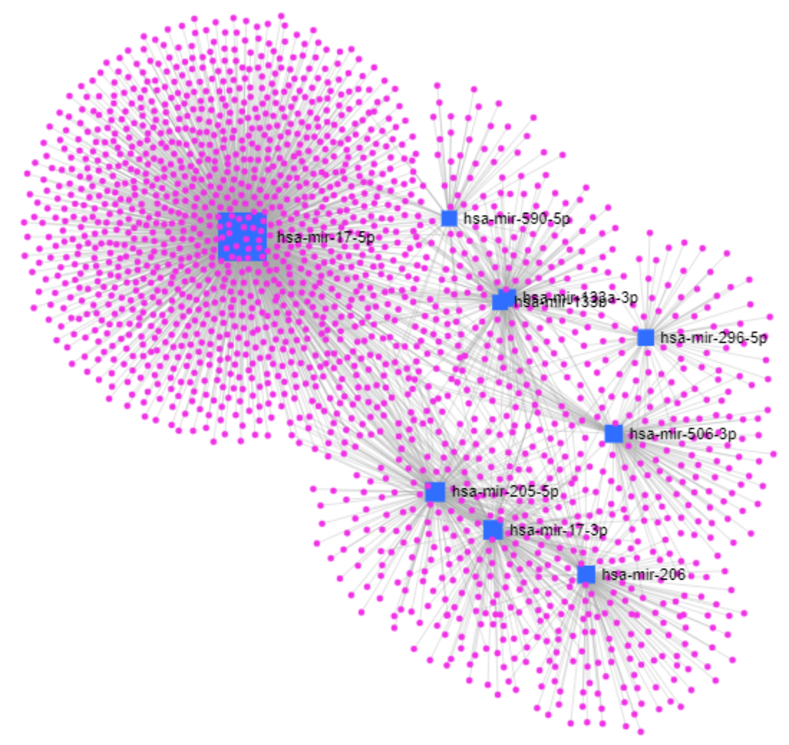

Supplement: S1 Fig — (TIF) [file pone.0315366.s002.tif]

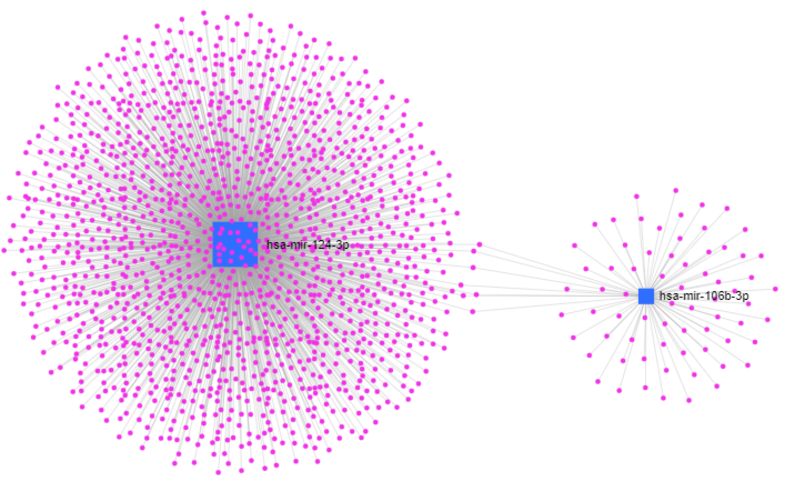

Supplement: S2 Fig — (TIF) [file pone.0315366.s003.tif]

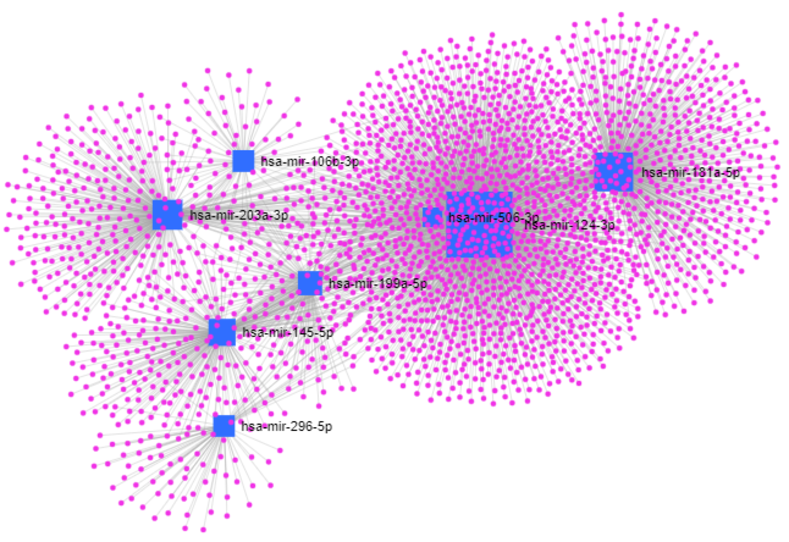

Supplement: S3 Fig — (TIF) [file pone.0315366.s004.tif]

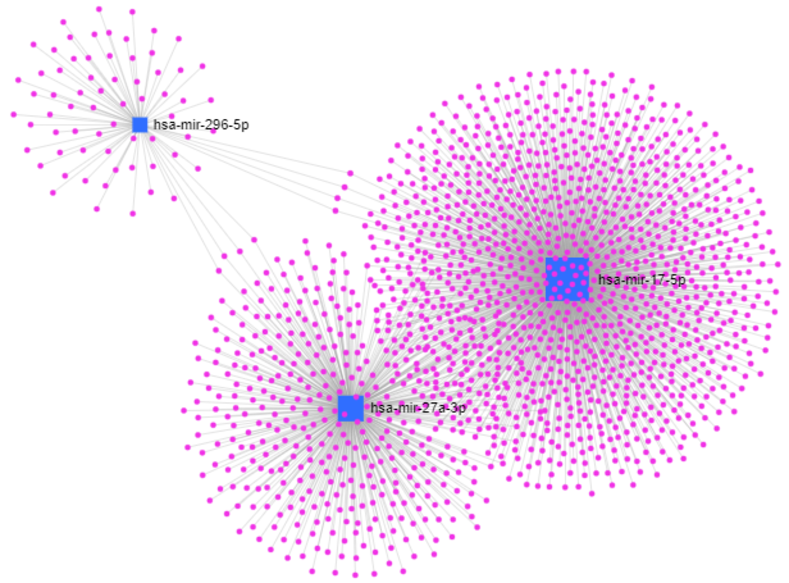

Supplement: S4 Fig — (TIF) [file pone.0315366.s005.tif]

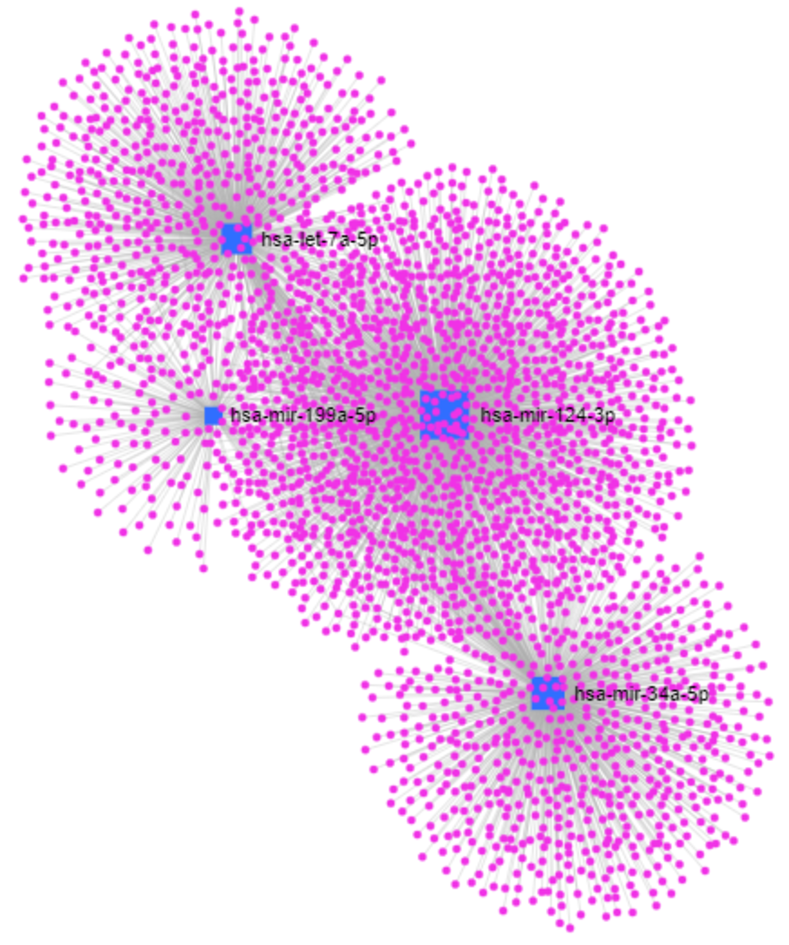

Supplement: S5 Fig — (TIF) [file pone.0315366.s006.tif]
